# Inflammation about the Third Molar

**Published:** 1888-03

**Authors:** E. G. Betty


// THE DENTAL REGISTER.

VOL. XLII.] MARCH, 1888. [No. 3.

### COMMUNICATIONS.

### Inflammations about the Third Molar.

Read before the Odontological Society of Cincinnati.

BY E. G. BETTY, D.D.S.

*Mr. President and Fellow Members:—*

I was very much astonished four days ago when our worthy Secretary, Dr. Fletcher, pounced in upon me with the very modest (?) request to prepare a paper upon this subject for the purpose of opening the discussion. Well, I forgive him, for he knew not what he did or he would not have so unmercifully floored a co-laborer at one fell swoop. In bringing this subject to your notice this evening I crave your indulgence with what must necessarily be but a fragment of a matter so broad in its bearings that it would take one a couple of years to study its specific and collateral literature. It implies far more than the caption would seem to indicate and we shall have to content ourselves with a bare mention of a few of the salient points which obtrude themselves upon us in our daily practice. As the topic seems to be confined to "Inflammations about the Third Molar," I take it to mean the lesions incident upon their eruption, and not those of the pulp and periosteum, they being common to all the teeth in the mouth. I purpose leaving out the consideration of inflammation as a pathological process and will merely say that the initial step in the progress of this phenomenon is irritation. We must then seek the irritant; which in this case you all naturally infer to be the third molar itself, erupting with all the complications of retardation, impaction, etc.

The irritation produced by the appearance (and very frequently the non-appearance of this organ) is very aptly and succinctly described by Dr. Abraham Robertson in an article on "Observations on the Lesions Occasioned by the Coming of the Wisdom Teeth" in vol. 5 of the *Cosmos*, pages 243 and 299, an article by the way it would well repay us all to study. He specifies six different ways in which the long train of disturbances occur.

*First*, "Like other teeth, by the irritation produced in coming through the gums. This is usually slight and temporary."

*Second*. If the space between the jaws at the ramus is very limited and the upper tooth precedes the lower, as often happens, it acquires its full length much sooner and the result is an impingement of this tooth upon the overlying gum of the lower; and in consequence bruising the part at every occlusion of the jaws. This is of but little moment and may be relieved by excision of the soft parts.

*Third*. When the space at the angle of the jaws is less than the combined length of the crowns of the two third molars, or, where one or other by priority of appearance has appropriated more than its due proportion of the territory.

It naturally follows that one is depressed and my observation has taught me that that one is the lower. As Dr. Robertson rightly says, the gum can form no attachment to the enamel, for the reason that it has no periosteum; consequently, the gum remains patulous, forming a ready receptacle for particles of food, saliva, epithelial casts, etc., which naturally decompose, the circumstances all being favorable. This putrefaction gives rise to inflammation, formation of pus, and, if proper treatment is not instituted the inevitable sequelle of necrosis and its attendant evils will ensue.

*Fourth*. "The wisdom tooth sometimes comes with its grinding or multi-cuspidated surface toward the cheek; when, if the cusps happen to be a little sharp, or if the tooth happens to become carious, thus rendering its edges sharp, it is very apt to produce excoriation and ulcerations of the cheek, and sometimes with the most serious consequences. This is particularly liable to be the case where the cheek is unusually fleshy and firm, as it is then brought into closer coaptation with the tooth."

This, gentlemen, you have often observed and, I doubt not, have at times been at your wits end to devise suitable means of extraction, for the condition is such that but little opportunity exists to turn the tooth outwards, none inwards on account of the solid palatine wall, and very frequently it is next to impossible to turn it backwards with an elevator. I mention this point because extraction is the manifest treatment, the removal of the irritant eventuating in the unassisted healing of the abscess or ulcerative patch as the case may be.

An item of great importance was overlooked by Dr. Robertson in this connection, viz.: the great liability of the irritation to the soft parts in this case, to result in malignant cancer in persons of middle and advanced life. The possibility of this is sufficient reason in my estimation to warrant severe measures in consummating extraction no matter how difficult it may be.

*Fifth.* "The crown of a lower wisdom tooth may turn inward, its grinding surface presenting to the base of the tongue, which it may excoriate in the same manner as one turning outward does the cheek."

*Sixth.* "Great disturbance is frequently caused, generally of a neuralgic character, by the grinding surface of the wisdom tooth (usually the lower) presenting against the posterior part of the second molar; which effectually stops the progress of its eruption. Other causes may have, to some extent, the same effect."

This half dozen ways in which the irritation manifests itself will, gentlemen, amply suffice to illustrate the manifold forms in which it occurs, and are sufficient for our purpose. We have now to consider some of the lesions incident upon, or rather, following the irritation thus set up. Usually the attention of the patient is called to the part by an uneasy feeling he can not describe, there is some slight pain, a more or less engorged condition of the surrounding soft parts and a disposition to bite or press upon the affected part in the endeavor to produce relief. This is common to the eruption of any tooth and if no complication exists, may be successfully treated by merely incising the superincumbent gum. The case becomes of graver import when the eruption of this tooth is delayed until late in life when the bony frame of the jaws has been thoroughly consolidated by extreme ossification and the absorption of the osseous structure more difficult by a surcharge of the system at large with the earthy salts. When in addition to this, the erupting tooth is a large one, (as a rule the lower one is such) the space at the ramus inadequate to accommodate it, and the other teeth firmly fixed as though welded together, we find the conditions favorable for severe trouble. We will have the crown deflected one way or another, in any direction but the right one, the consequence being that eruption is retarded at first and finally as the root develops, the crown being unable to adjust itself properly, impaction results as a legitimate outcome of the circumstances. The symptoms which now develop, may manifiest themselves in a great variety of ways just as the conditions are variable, and there is no telling at the start what turn affairs will take. Should the eruption be retarded merely, the local lesions will be of an inconsequential character. Pain, heat due to an accelerated flow of blood to the part, some œdema, usually confined to the immediate neighborhood of the tooth. These symptoms may subside without any interference, either surgical or therapeutic. They may recur at intervals of a few weeks or months with decreasing severity, until finally the tooth takes its proper place in the arch or a little to one side of the line. Retardation may also assume a more severe phase in the character of the local lesions. The pain may be excessive, the œdema extend to the cellular tissue of the muscles of the cheek and throat, a slight tonsilitis occur. The parts become hard and the rigidity often renders deglutition difficult, to say nothing of its becoming so painful as to render it impossible. Infiltration takes place in many cases, the inspissation prolonging the immobility of the muscles. Pus may be in process of formation during this time and may discharge itself at any point.

The constitutional symptoms include fever, loss of appetite, constipation, insomnia, mental perturbation.

The treatment in such cases is without a doubt, extraction. This of course is difficult at the extreme stage of the case and would better be done at once, if other measures such as hot fomentations, excision, depletion, etc., have failed to afford any amelioration of the suffering of the patient. The rigidity of the muscles operating the jaws may sometimes be reduced by warm fomentations long continued, together with thorough cartharsis; a chologogue, blue mass for instance, at night, say 10 grains, followed in the morning with an ounce of Epsom salts in as much water as the patient can manage to swallow. Should there be no other result, the fever will be reduced and the general tone of the system improved by this cleansing of the alimentary tract. To tell the truth, it should be done in the first instance. If this treatment has effected sufficient or partial relaxation, remove the offending organ in the best manner possible under the circumstances; they will naturally suggest themselves to the inventive operator, even if he is obliged to extemporise a suitable instrument to meet the emergency.

There are cases in which this course of treatment is ineffectual, an examination even, impossible without the administration of an anæsthetic. Nitrous oxide is contra-indicated, it usually producing rigidity and would be worse than useless in most instances. Chloroform produces the greatest relaxation of muscular tissue and is preferable. A deep incision must be made at the point where the tooth is expected to be and if it is found, removal is indicated even if the surgeon must be called in to operate by cutting through the cheek at the ramus and trephining.

So far as I am able to learn, this procedure is generally deferred to the last moment, when the constitutional symptoms have become grave and the patient is threatened with extensive necrosis of the bony surroundings, pyæmia, and what not.

The lower jaw is usually the seat of impaction, though the upper sometimes exhibits grave cases of retardation, impaction, development of the tooth in the antrum, that part being complicated seriously, oftentimes.

During the progress of a complicated eruption, the adjacent parts about the mouth may become involved in inflammations more or less extensive. Cellutitis may extend as far down as the shoulder, rigidity of the muscles throughout the region of the neck. The tonsils may become inflamed and suppurate—a grave complication when we remember that the internal carotid is next door neighbor and the immobility of the muscles of the jaw prevents the operator, either dentist or surgeon, from opening the tonsil and discharging the pus. There may also be more or less extensive inflammation of the mucus membrane of the mouth, fauces, and pharynx. The lining membrane of the eustachian tube may convey the inflammation into the ear, a complication also of grave import.

Another point I wish to bring to your notice is that an erupting third molar is often responsible for the obscure facial neuralgias we are called upon at times to treat. As Trudeau (a French writer of twenty-five or thirty years ago) says, it is the pressure of the developing root upon some nerve fiber which causes the neuralgias.

We know well enough now that pressure upon a nerve filament at any point produces pain oftentimes at a situation far remote from the seat of trouble and we call it reflex.

Thus you see, gentlemen, from the mention I have made of a few only, of the possibilities, how broad this subject is, how great the varieties and variations of the symptoms are and how difficult it is to make a correct prognosis of any case when seen in the initial stage.

So far as treatment is concerned, I invariably incise upon sight and if the gum is large, bruised upon occlusion, or a cusp has appeared, I excise liberally. Counter-irritation and the topical application of anodynes is palliative at best and of little real value. Cathartics at the beginning do more good than if administered later on when the constitutional disturbance has become well marked and rigidity of the local surroundings has seriously interfered with surgical treatment. The latter after all is the only effective method of dealing with such cases and it will not pay to dilly-dally, hoping to afford relief until the tooth has appeared. We run the risk of a serious case and it is far better to go in heroically at the outset.

I had intended when I began this sketch, to make a few remarks upon the advisability of extracting the second molar to facilitate the eruption of the third; also upon the theory of the gradual disappearance of the third molar from the civilized races; its far superior development in the lower races, etc., but concluded that I had already undertaken more than I could well handle.

With these crude remarks upon this most interesting topic, gentlemen, I shall turn it over to you to cuss, and discuss, the former I doubt not you have all been tempted to do, and the latter I earnestly hope you will do.

